# Modified biochar from Moringa seed powder for the removal of diclofenac from aqueous solution

**DOI:** 10.1007/s11356-019-06844-x

**Published:** 2019-12-28

**Authors:** Afrouz Bagheri, Emmanuel Abu-Danso, Jibran Iqbal, Amit Bhatnagar

**Affiliations:** 1 Department of Environmental and Biological Sciences, University of Eastern Finland, FI-70211 Kuopio, Finland; 2 College of Natural and Health Sciences, Zayed University, P.O. Box 144534, Abu Dhabi, United Arab Emirates

**Keywords:** *Moringa oleifera* seed powder, Biochar, Adsorption, Diclofenac, Phosphate modification

## Abstract

In this study, Moringa seed powder (M_SP_) was pyrolyzed at 450 °C to synthesize Moringa seed powder biochar (M_SP_B) and treated with phosphoric acid (H_3_PO_4_) to synthesize phosphate-modified Moringa seed powder biochar (M_SP_B-HPO) as an adsorbent for the removal of diclofenac (Dfc) from aqueous solution. Fourier transform infrared (FTIR) analysis, energy dispersive X-ray spectroscopy (EDS), scanning electron microscopy (SEM), and pH point of zero charge (pH_pzc_) were conducted to give more insight into the adsorbent’s properties. The SEM analysis showed the transformations in the surface morphology from the parent material to the synthesized materials after the thermal and acid treatment. EDS analysis revealed the variation in the elemental composition of the materials prior to and after adsorption of Dfc ions. The FTIR analysis showed changes and peak intensities of functional groups involved in Dfc removal. The pH_pzc_ showed the charge carried by M_SP_B-HPO in different pH conditions. Isotherm data best matched the Sips model, and the pseudo-second-order model best described the adsorption kinetics. The maximum adsorption capacity of M_SP_B-HPO by Sips model was found to be 100.876 mg g^−1^.

## Introduction

According to international scientific statistics, 70% of accessible fresh water is utilized in agriculture and food industry (Wong et al. 2018). Water demand is increasing, while the quality and quantity of freshwater are continuously decreasing due to anthropogenic activities (Wong et al. 2018; Murtaza et al. 2019). In recent decades, numerous micropollutants including pharmaceuticals have emerged in different environments. Pharmaceutical compounds have found their way into freshwater resources and consequently, decrease the quality. Diclofenac (Dfc), which is used as an analgesic, is one of the main priorities of emerging contaminants. It requires special treatment due to the noxious effects on environmental health (Moreno et al. 2009; Beyki et al. 2017; Barczak et al. 2018; Li and Yang 2018; Sayed et al. 2019). Removal processes of pharmaceutical contaminants such as photocatalytic degradation, biological treatment, and filtration have been extensively used. These processes have some level of efficiency, but they have their limitations such as extended contact time, high operational, and energy cost. Adsorption, however, has many advantages over the different techniques including vast efficiency, easiness, and low operational cost. In adsorption process, contaminants are adsorbed from the liquid phase to a solid phase (Beyki et al. 2017; Barczak et al. 2018; Li et al. 2018).

Biomass is a significant resource that can be converted to a carbonized biochar, a bio-material that has received wide attention. Biochar is carbon-rich microporous material and has high-value application in water pollution remediation (Jindo et al. 2014). *Moringa oleifera* is a tropical and subtropical plant species with a good yield of seeds. The other parts such as the leaves have been used as sorbent in various water treatment studies (Maina et al. 2016). The seed husk has also been studied in the adsorption of diclofenac (Araujo et al. 2018), acid black 1 and basic red 2, reactive dyes, heavy metals, atrazine, and nitrobenzene from solution (Buildings et al. 1997; Akhtar et al. 2007; de Carvalho et al. 2015; Matouq et al. 2015; Tavengwa et al. 2016; Maina et al. 2016; Souza et al. 2016; Tavares et al. 2017; Coldebella et al. 2017; Khorsand et al. 2017; Maria et al. 2018; Shirani et al. 2018).

In this study, a simple technology was used to prepare phosphate-modified *Moringa oleifera* seed powder biochar (M_SP_B-HPO) using mild phosphoric acid (0.5 M) as phosphate source and used for the adsorption of diclofenac (Dfc) from water. *Moringa**oleifera* seed powder biochar (M_SP_B) was synthesized at 450 °C in a N_2_ injection chamber. The prepared biochar was spun in phosphoric acid to synthesize M_SP_B-HPO adsorbent. Parameters including effect of pH, initial Dfc concentrations, and contact time on the adsorption of Dfc by M_SP_B-HPO were investigated. Characterization of M_SP_B-HPO as well as the adsorption behaviors was studied to evaluate the performance of their practical applications in Dfc removal from water.

## Materials and methods

### Chemicals

*Moringa oleifera* seeds were obtained commercially. Sigma-Aldrich (Suomi) supplied sodium chloride (NaCl 99%) and sodium hydroxide (NaOH 98.9%). Phosphoric acid (85 wt %) and diclofenac sodium salt (98.5%) were purchased from Acros (Geel, Belgium).

### Thermal treatment of Moringa seeds powder (M_SP_)

The M_SP_B was produced by thermal pyrolysis. The sample was placed in a crucible and then placed in a fixed-bed stainless steel tubular furnace under N_2_ atmosphere. The temperature was raised from room temperature to 450 °C at a heating rate of 10 °C/min and sustained for ca. 2 h. The biochar was allowed to cool and then ground and sieved through a 160-μm sieve.

### Modification of Moringa seeds powder biochar (M_SP_B)

An amount of M_SP_B (ca. 1.5 g) was pulverized and mixed with 50-mL 0.5 M phosphoric acid (H_3_PO_4_) and spun at 80 rpm speed for 24 h. M_SP_B-HPO was washed using deionized water to neutral pH and dried overnight in an oven at 40 °C.

### Characterization

The surface morphology of M_SP_ and the biochars were analyzed with Zeiss sigma HDVP (Carl Zeiss GmbH, Oberkochen Germany) scanning electron microscopy. Separate voltages and magnifications were chosen to optimize the image. Samples for the analysis were sputtered with gold by using agar auto gold sputter. Elemental composition of the synthesized materials was analyzed with energy-dispersive X-ray spectroscopy (EDS) (Sigma HDVP, Carl Zeiss GmbH, Germany). The Fourier transform infrared (FTIR) analysis of the different materials in this study was recorded between 400 and 4000 cm^−1^ at 32 scans using Thermo Nicolet Nexus 8700 model (Thermo electron, Madison USA) to examine the changes in functional groups on the synthesized materials, before and after adsorption.

### Adsorption experiments

Batch adsorption experiments were performed to investigate diclofenac adsorption. A solution of 100 mg L^−1^ (stock) was prepared and covered to prevent photo-degradation. Dilution was used to prepare different concentrations of Dfc (2.5–70 mg L^−1^) from the stock solution. A predetermined quantity of M_SP_B-HPO and a volume of 40 mg L^−1^ concentration of Dfc (10 mL) at (pH ~ 5) were both put in capped falcon tubes and were agitated at 80 rpm on a shaker at room temperature until equilibrium time. After equilibration time, M_SP_B-HPO was filtered from Dfc solution using filters with 0.45 μm pore size (Sartorius, Gmbh Germany). The Dfc residual concentrations of all batch adsorption experiments were analyzed with UV-VIS Spectrophotometer (UV-2401 PC (double beam)) at λ_max_ = 287 nm wavelength. The adsorbed Dfc at equilibrium capacity onto the M_SP_B-HPO was analyzed using eq. (1) and the percentage removal efficiency was determined according to eq. (2) (Daneshvar et al. 2012):

1 $$ {q}_e=\frac{\left( Ci- Ce\right)V}{m} $$

2 $$ R\ \left(\%\right)=\frac{\left( Ci- Ce\right)}{Ci}\times 100 $$

where *q*_e_ is the adsorption capacity of M_SP_B-HPO (mg g^−1^), *C*_i_ and *C*_e_ are the initial and final diclofenac concentrations (mg L^−1^), *v* is the volume of the diclofenac (L), *m* is the amount of M_SP_B-HPO (g), and *R* (%) is the removal efficiency.

## Experimental results and discussion

### Scanning electron microscopy analysis

**Fig. 1:**
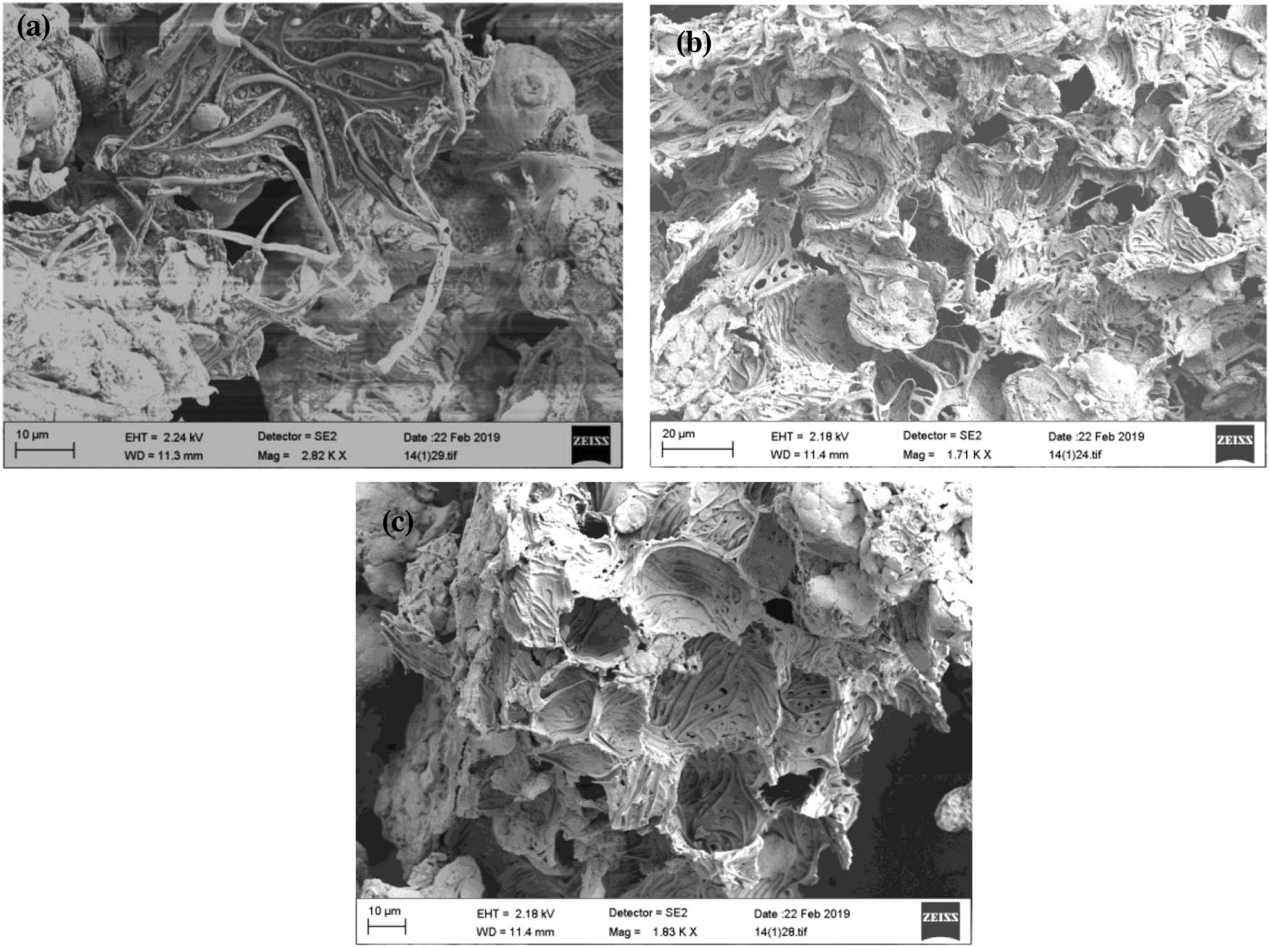
SEM images of **a** M_SP_, **b** M_SP_B, **c** M_SP_B-HPO

The microscopic morphological observations of M_SP_, M_SP_B, and M_SP_B-HPO are presented in Fig. 1a–c. The micrograph of M_SP_ (Fig. 1a) showed a non-uniform complex fiber matrix with no particular shape as reported elsewhere (Tavengwa et al. 2016). However, after the thermal treatment (450 °C) in a N_2_ environment (Fig. 1b), the surface morphology revealed macropores and irregular trough-like patterns. The structure appeared frail and the cell morphology of plant biochar was absent. The phosphate-modified biochar (M_SP_B-HPO) revealed trough-like patterns; however, it also showed cup-like cell shapes with embedded macropores (Fig. 1c). Compared to M_SP_B, M_SP_B-HPO showed well-defined edges which suggests further cleaving of the material from the M_SP_B. As seen from Fig. 1c, the chemical modification by H_3_PO_4_ could enhance the specific internal surface area (Chen et al. 2017).

### Energy-dispersive X-ray spectroscopy (EDS) analysis

The elemental composition of the synthesized materials (atomic %) was analyzed by EDS. As the results illustrate in Table 1, the chemical composition of biochar changes significantly during biomass pyrolysis and the subsequent modification by weak H_3_PO_4_. The EDS results showed that the atomic percentages of C and O in the M_SP_, M_SP_B-HPO on one hand, and M_SP_B-HPO after Dfc adsorption were 73.2%, 22.2%; 84.9%, 8%; 87.3%, 8.3%; and 89.8, 9.2%, respectively. After thermal induction, the decarboxylation and dehydration of Moringa biomass into H_2_O, CO_2_, CO, etc. occurred and a larger amount of O compared to C was lost, because electrons during destruction of carbonyl groups move to oxygen and formed radicalized oxygen. In addition, biomass generally contains some removable O fractions while after pyrolysis, some still remains in the biochar (Harvey et al. 2012; Rutherford et al. 2012; Jindo et al. 2014; Chen et al. 2017). The P content of M_SP_B-HPO and M_SP_B-HPO after Dfc adsorption was 1.5 and 1.3%, respectively, suggesting that phosphates was successfully etched on M_SP_B.

**Table 1:** Elemental (atomic %) analyses of M_SP_, M_SP_B, M_SP_B-HPO, and M_SP_B-HPO/Dfc

Element	M_SP_	M_SP_B	M_SP_B-HPO	M_SP_B-HPO/Dfc
C	73.2	84.9	87.3	89.8
N	8.2	7	7.3	9
O	22.2	8	8.3	9.2
P	0	0	1.5	1.3
Cl	0	0	0	0.3

### Point of zero charge (pH_pzc_) analysis of M_SP_B-HPO

**Fig. 2:**
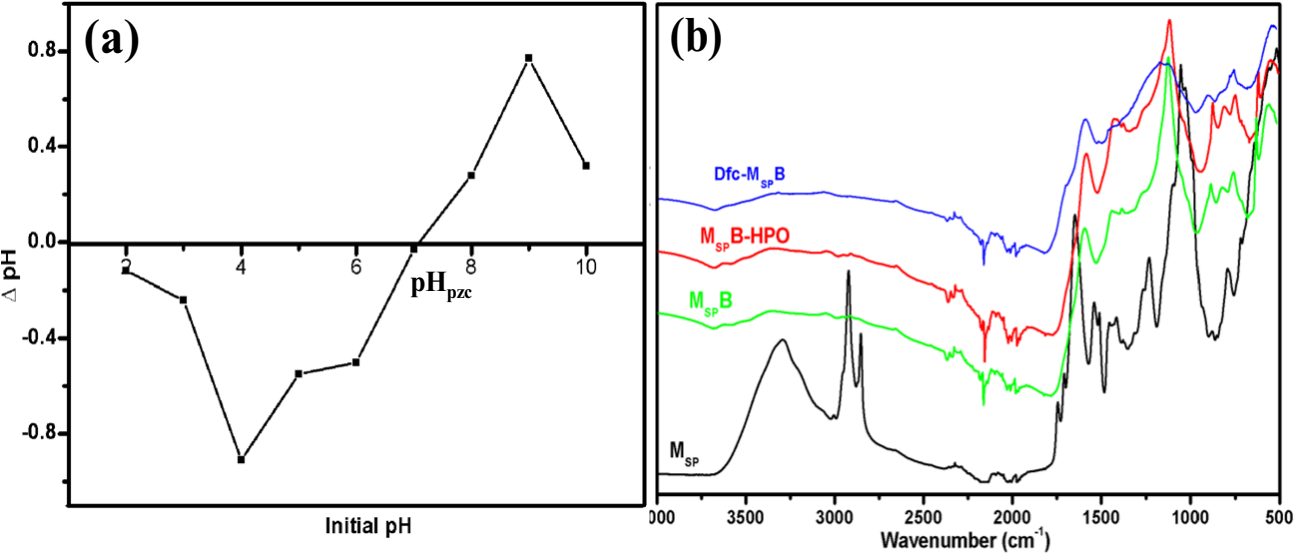
**a** pH_pzc_ of M_SP_B-HPO, **b** FTIR spectra of M_SP_, M_SP_B, M_SP_B-HPO, and Dfc-M_SP_B

The pH_pzc_ analysis of M_SP_B-HPO was studied from 2 to 10 pH range and the result is presented in Fig. 2a. The pH_pzc_ was found to be 7.09 and the result showed that the M_SP_B-HPO has variable electrostatic charges in different pH conditions. In acidic medium, the material was negatively charged; however, the charge changed to positive when the material was studied in alkaline medium. This characteristic of M_SP_B-HPO suggests that it can have a favorable interaction with a positively charged adsorbate in an acidic medium.

### Fourier transform infrared spectroscopy analysis

The different infrared spectrum of the different materials is presented in Fig. 2b. The main peaks on the M_SP_ were found at ~ 3300, 2923, and 1007 cm^−1^ which represent –OH, –CH, and –OCH_3_ functional groups, respectively, as found in other study (Ramavandi 2014). The peaks assigned to carbonyl (C=O) functional groups were found at ~ 1635, ~ 1700, and 1735 cm^−1^ (Ramavandi 2014). The peaks of some aforementioned functional groups shifted after thermal treatment of M_SP_. This phenomenon can be attributed to the thermal assisted destruction or conversion of the functional groups. The peak at ~ 1730 cm^−1^ shifted on M_SP_B spectra because C=O is easy to be lacerated after heat treatment. After the modification to form M_SP_B-HPO, two peaks associated with phosphate and carbon interaction appeared, firstly at ~ 1095 cm^−1^ assigned to P–O–C stretching vibration mode (Coates et al. 2000)^;^ this peak, however, significantly changed after Dfc adsorption. The other peak representing (P–O) bond at 745–725 cm^−1^ appeared (Pavia et al. 2009). The FTIR of M_SP_B-HPO after Dfc adsorption (Dfc-M_SP_B) showed a reduction in the –P bond after diclofenac adsorption.

### Adsorption of diclofenac by M_SP_B-HPO

#### Effect of pH on the adsorption of Dfc by M_SP_B-HPO

**Fig. 3:**
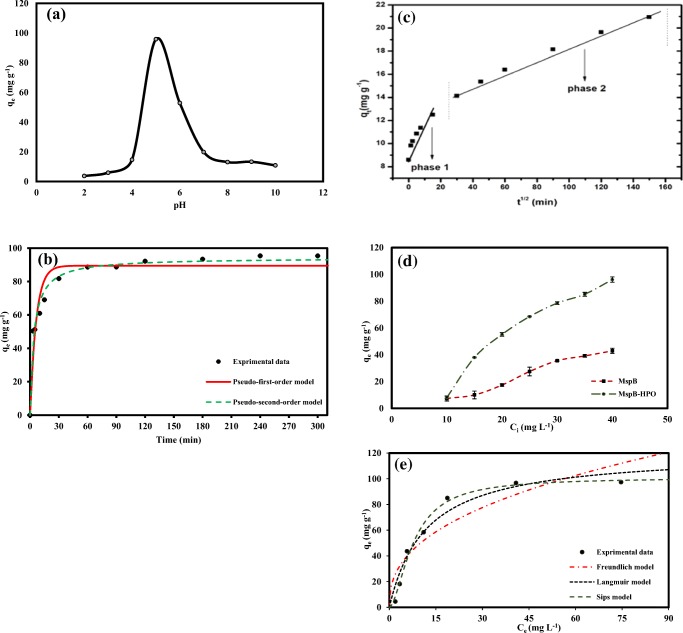
**a** Effect of pH on adsorption of Dfc (40 mg L^−1^) by MS_PB_-HPO (0.4 g L^−1^), **b** adsorption kinetic data modeling of Dfc (40 mg L^−1^) by M_SP_B-HPO (0.4 g L^−1^), **c** intra-particle diffusion modeling, **d** comparative adsorption of of Dfc (10–40 mg L^−1^) by M_SP_B and synthesized M_SP_B-HPO (0.4 g L^-1^), and **e** adsorption isotherm data modeling of Dfc (2.5–100 mg L^−1^) by M_SP_B-HPO (0.4 g L^−1^)

**Fig. 4:**
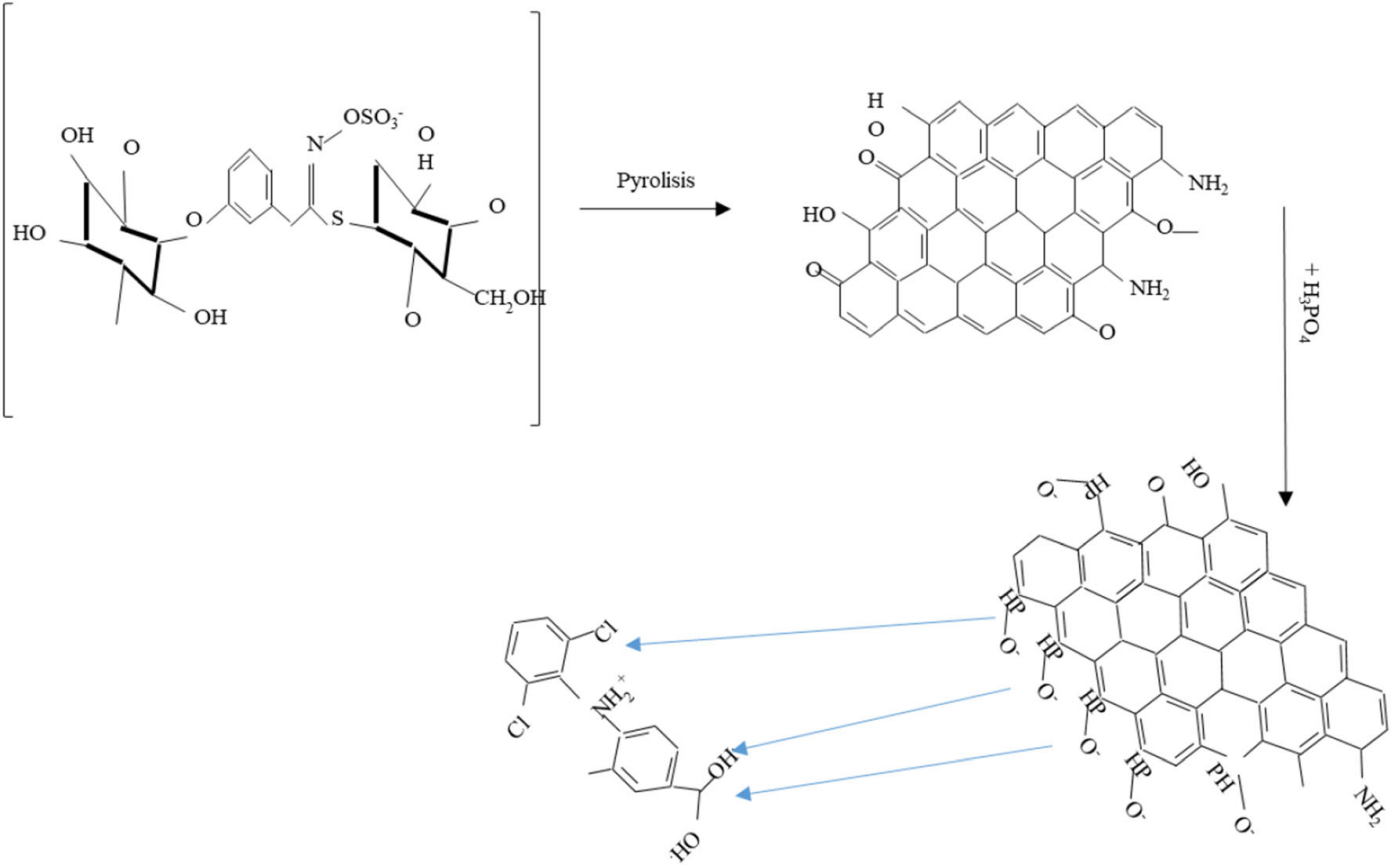
Plausible reaction scheme of conversion of M_SP_ (Jaja-chimedza et al. 2017) to biochar, negatively charged synthesized M_SP_B-HPO and adsorption of Dfc

The influence of pH on the adsorption of Dfc was investigated in pH range of 2–10. To adjust the solution pH, known concentrations of hydrochloric acid and sodium hydroxide were used and the results are shown in Fig. 3a**.** The results revealed higher adsorption capacities in moderate acidic pH. The highest removal capacity of 95.85 mg g^−1^ representing 82.8% removal efficiency was recorded at pH ~ 5. The adsorption capacity decreased sharply beyond pH ~ 5 and the reduction in adsorption capacity continued in basic pH. The significant decrease in the adsorption capacity in basic medium can be attributed to the rapid deprotonation in the system which results in repulsion between the M_SP_B-HPO surface thereby preventing complexation of M_SP_B-HPO surface and the Dfc ions as reported in other study (Hu and Cheng 2015). During the modification process, phosphates from dissociated H_3_PO_4_ interact with the graphite-like crystallites of the biochar. This interaction results in P–O–C linkages on the modified biochar to form a net negatively charged surface as (Wang et al. 2017). The synthesized M_SP_B-HPO as a carbonaceous material is able to adsorb the pH modified Dfc via π–π interactions and these interactions are controlled by the changes in pKa of Dfc because changes in the pKa values can affect Dfc to take on cationic, neutral, or anionic character (Jiang et al. 2015; Lonappan et al. 2018). Furthermore, low-temperature synthesized biochars including M_SP_B are hydrophobic and adsorb pollutants effectively in an acidic phase (Fig. 4).

#### Adsorption kinetic modeling

The influence of contact time on M_SP_B adsorption of Dfc ions was performed to obtain data on the overall uptake rate of adsorbate with time (Shirani et al. 2018). The experiment was done using 40 mg L^−1^ Dfc concentration and 0.4 g L^−1^ dose of M_SP_B and studied from 1 to 300 min. The results (Fig. 3b) showed that the adsorption capacity increased rapid initially (0–30 min), then increased in a slow rate and then stabilized to reach equilibrium. At equilibrium, the unavailability of free active adsorption sites resulted in no further adsorption. Adsorption capacity and removal efficiency reached 95.383 mg g^−1^ and 83%, respectively. Two kinetic models were used to evaluate the kinetic data as reported in other study (Iqbal et al. 2019).

Pseudo-first-order (PFO) model (Eq. (3)) (Ho 2004).

3 $$ {q}_t={q}_e\left(1-{e}^{-{k}_{1t}}\right) $$

Pseudo-second-order (PSO) model (Eq. (4)) (Ho and Mckay 1999).

4 $$ {\mathrm{q}}_{\mathrm{t}}=\frac{{\mathrm{k}}_2{\mathrm{q}}_{\mathrm{e}}^2\mathrm{t}}{1+{\mathrm{k}}_2{\mathrm{q}}_{\mathrm{e}}\mathrm{t}} $$

The intra-particle diffusion data was analyzed as reported elsewhere (Samiey 2015) using Eq. (5) which is written as

5 $$ {\mathrm{q}}_{\mathrm{t}}=\mathrm{I}+{\mathrm{k}}_{dif}{t}^{0.5} $$

where *q*_e_ and *q*_t_ are the adsorption capacity (mg g^−1^) at equilibrium and time *t* (min), respectively, and *k*_1_ (min^−1^) and k_2_ (g mg^−1^ min^−1^) represent rate constants of PFO, and PSO. I (mg g^−1^) is the intercept of the boundary layers thickness and k_*dif*_ (mg g^−1^ min^−0.5^) is intra-particle diffusion rate constant.

The summary of adsorption kinetic data evaluated by the three models is presented in Table 2. The values of the model parameters suggest that the PSO model best fitted the kinetics data (*R*^2^ = 0.989; RMSE = 3.554) compared to the other studied models. The differences in the experimental (*q*_e exp_) and calculated maximum adsorption capacities (*q*_e cal_) for the models used in the analysis also suggest that the PSO best fitted the adsorption kinetics. The fitting of the experimental data to the PSO kinetics model suggests a chemical process involving ionic exchanges between the pH modified Dfc and the M_SP_B-HPO surface.

**Table 2:** Adsorption kinetics parameters and data of Dfc removal by M_SP_B-HPO

Kinetic model	Parameter	Data
Pseudo-first-order	*q*_e exp_ (mg g^−1^)	95.383
	*q*_e cal_ (mg g^−1^)	89.474
	*k*_1_ (min^−1^)	0.155
	RMSE	6.674
	*R*^2^	0.964
Pseudo-second-order	*q*_e exp_ (mg g^−1^)	94.383
	*q*_e cal_ (mg g^−1^)	94.206
	*k*_2_ (g mg^−1^ min^−1^)	0.002
	RMSE	3.554
	*R*^2^	0.989
Intra-particle diffusion	*q*_e exp_ (mg g^−1^)	94.383
	k_*dif*_ (mg g^−1^ min^−0.5^)	3.161
	I (mg g^−1^)	50.950
	RMSE	7.666
	*R*^2^	0.961

From the intra-particle diffusion modeling, the movement of the Dfc ions occurred in two different phases which were an initial rapid phase and a slow and stabilized phase as shown in Fig. 3c. These results suggest that diffusion was probably not the rate-limiting step but other factors may have control on the rate of adsorption (Yakout and Elsherif 2010; Abu-Danso et al. 2018).

#### Dfc adsorption isotherm studies

Different initial concentrations of Dfc (10 to 40 mg L^−1^) and its effect of on the adsorption was investigated with a constant amount of M_SP_B-HPO (0.4 g L^−1^). The adsorption capacity of unmodified M_SP_B was also examined simultaneously (Fig. 3c)**.** The results revealed that adsorption increased with an increase in Dfc concentration for both adsorbents. However, the removal capacity of M_SP_B-HPO was found to be two times higher than M_SP_B (96.11 mg g^−1^ and 42.8 mg g^−1^) at equilibrium adsorption which suggests that adsorption efficiency increased significantly after modification**.**

#### Adsorption isotherm modeling

Isotherm systems describe interaction between adsorbate and adsorbent. Overall, an isotherm curve demonstrates the phenomenon of the retention from the aqueous phase to a solid phase (Foo and Hameed 2010; Ahmed 2017). To analyze the experimental data, three isotherm models were used to elucidate how Dfc ions are adsorbed onto the surface of M_SP_B-HPO. The Sips model (Sips 1948) is a two-parameter model that combines characteristics of Freundlich and Langmuir models. The description of lower concentration adsorption isotherm data by the Sips model is similar to the Freundlich model and it occurs in a heterogeneous layer, whereas a monolayer adsorption takes place at higher adsorbate concentrations similar to the assumption of Langmuir model (Noori et al. 2017).

The Sips model is written as

6 $$ {\mathrm{q}}_{\mathrm{e}}=\frac{{\mathrm{q}}_{\mathrm{m}{\left({\mathrm{K}}_{\mathrm{s}}{\mathrm{C}}_{\mathrm{e}}\right)}^{\mathrm{m}}}}{1+{\left({\mathrm{K}}_{\mathrm{s}}{\mathrm{C}}_{\mathrm{e}}\right)}^{\mathrm{m}}} $$

where *q*_e_ (mg g^−1^) and *C*_e_ (mg L^−1^) are the adsorption capacity and Dfc concentration at equilibrium time, respectively, *m* is the exponent that is between 0 and 1, and the Sips affinity is denoted by K_S_ (L mg^−1^).

Adsorption that follows Langmuir model occurs on definite adsorption sites by ions with similar equilibrium adsorption constants. The Langmuir model predicts monolayer adsorption (Langmuir 1918; Rathod et al. 2015). For an adsorption process that follows Langmuir model, the adsorbed ions are attached onto definite adsorption sites with similar energy. Langmuir model can be written as Eq. (7) (Langmuir 1918):

7 $$ {\mathrm{q}}_{\mathrm{e}}=\frac{{\mathrm{q}}_{\mathrm{m}{\mathrm{K}}_{\mathrm{L}}{\mathrm{C}}_{\mathrm{e}}}}{1+{\mathrm{K}}_{\mathrm{L}}{\mathrm{C}}_{\mathrm{e}}} $$

where *K*_L_ (L mg^−1^) is the Langmuir constant.

Freundlich model is an empirical model which describes interplay between multilayer and non-ideal sorption on a heterogeneous surface (Freundlich 1909; Rathod et al. 2015). The equation is defined as

8 $$ {\mathrm{q}}_{\mathrm{e}}={\mathrm{K}}_{\mathrm{F}}{\mathrm{C}}_{\mathrm{e}}^{1/\mathrm{n}} $$

where K_F_ (mg g^−1^) is the Freundlich affinity constant and *n* (g L^−1^) is the heterogeneity factor.

The modeled isotherm data are shown in Fig. 3d. The root mean square error (RMSE) and the correlation coefficients (*R*^2^) obtained for the Sips model (2.842) and (0.993) as well as the Langmuir model (5.493) and (0.979), respectively, are presented in Table 3. The results suggest that the adsorption isotherm followed Sips and Langmuir models compared to the other studied model. This form of adsorption has been reported in other study (Zito et al. 2015). However, comparing the two models, a stronger correlation was found with the Sips model over the Langmuir although the process was studied under similar energy (room temperature). This phenomenon can likely be assigned to the mode of equilibrium adsorption determination at a single temperature by the two models since the parameters of determination vary between them. The results show that M_SP_B-HPO adsorbs Dfc as a monolayer; however, other adsorption surfaces were also available and contributed to the adsorption process, hence, the description by Sips model. This type of adsorption has been reported in other study (Daneshvar et al. 2018). The highest Sips adsorption capacity was found to be 100.876 mg g^−1^.

**Table 3:** Isotherm model data for the removal of Dfc by M_SP_B-HPO

Isotherm model	Parameter	Data
Sips	*q*_e exp_ (mg g^−1^)	97.406
	*q*_e_ (mg g^−1^)	100.876
	m	0.586
	*K*_S_ (L mg^−1^)	0.030
	*R*^2^	0.993
	RMSE	2.842
Langmuir	*q*_e exp_ (mg g^−1^)	97.406
	*q*_m_ (mg g^−1^)	121.112
	K_L_ (*L* mg^−1^)	0.085
	*R*^2^	0.979
	RMSE	5.493
Fruendlich	K_F_ (mg g^−1^)	19.751
	n (g L^−1^)	2.485
	*R*^2^	0.915
	RMSE	10.100

#### Effect of co-existing cations

**Fig. 5:**
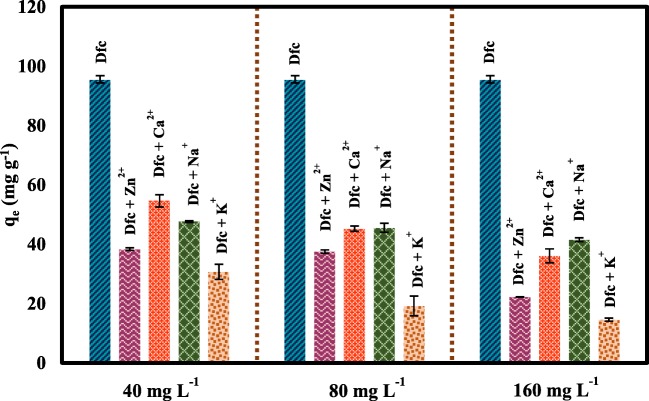
Effect of competing ions on Dfc (40 mg L^−1^) removal by M_SP_B-HPO (0.4 g L^−1^)

The influence of co-existing cations on the Dfc removal by M_SP_B-HPO was studied in with 40, 80, and 160 mg L^−1^ concentrations of Zn^2+^, Ca^2+^, Na^+^, and K^+^ cations in a constant Dfc (40 mg L^−1^ (pH ~ 5)) concentration. As shown in Fig. 5, M_SP_B-HPO’s removal capacity for Dfc in the presence of these common cations generally decreased when the cation concentrations increased. The impact of the presence of K^+^ and Zn^2+^ strongly interfered with Dfc adsorption than the other studied cations. This trend of adsorption in which the adsorption capacity reduces in the presence of cations can be attributed to their chemical behavior. The presence of cations can result in a repulsion with Dfc which inhibit the mass transfer coefficient from the solution to the sorbent (Abu-danso et al. 2019; Abujaber et al. 2019). On the other hand, the ionic strength of Dfc solution can decrease the adsorption capacity of M_SP_B-HPO through electrostatic interactions (Martinez-costa et al. 2018; Rafi et al. 2018). Moreover, when the concentration of cations was increased from 40 to 160 mg L^−1^, the removal of Dfc decreased considerably.

#### Regeneration of M_SP_B-HPO

**Fig. 6:**
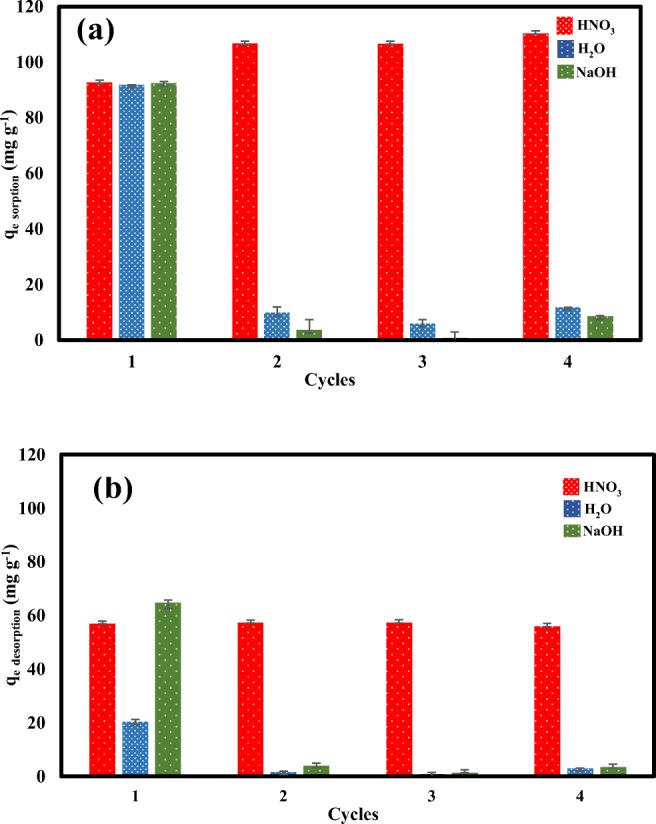
Regeneration studies of Dfc removal by M_SP_B-HPO **a** adsorption and **b** desorption

Reusability of M_SP_B-HPO in repeated Dfc removal was studied using deionized H_2_O, 0.1 M HNO_3_, and 0.1 M NaOH during four regeneration cycles and the results are presented in Fig. 6 a and b. Reusability studies are conducted to test the resilience of the synthesized material and also save water treatment cost. The results showed that, after 4 cycles, the synthesized M_SP_B-HPO still had adsorption capacity > 100 mg g^−1^ under acidic condition (Fig. 6a). The limitation of the other used eluents in eluting the Dfc ions could be due to the unfavorable alkaline conditions because the Dfc was adsorbed in weak acidic conditions. Both adsorption and desorption trend in the study suggest that MSPB-HPO is regenerated reasonably after waste water treatment.

## Conclusions

In this study, phosphate-modified Moringa seeds biochar (M_SP_B-HPO) was synthesized from *Moringa oleifera* seeds biochar (M_SP_B) with phosphoric acid as the phosphate source. The synthesized M_SP_B-HPO was used for the removal diclofenac from aqueous medium. Different characterization techniques such as SEM, EDS, and FTIR were used to analyze both raw and the modified materials. The SEM image revealed that the synthesized M_SP_B-HPO was a porous material. FTIR showed that (P-CH_3_) and (P-O) bonds appeared after modification. In addition, pH_pzc_ showed the charge carried by the M_SP_B-HPO in acidic and basic media. The batch adsorption experiment results displayed the removal capacity of Dfc onto the M_SP_B-HPO. The pseudo-second-order model could best describe the adsorption kinetics. Sips model compared to the other models described the isotherms data better, and the maximum Sips adsorption capacity was found to be 100.876 mg g^−1^. The study showed that M_SP_B-HPO is recyclable for the removal of Dfc from aqueous environments.
